# Granule-Dependent Natural Killer Cell Cytotoxicity to Fungal Pathogens

**DOI:** 10.3389/fimmu.2016.00692

**Published:** 2017-01-11

**Authors:** Henry Ogbomo, Christopher H. Mody

**Affiliations:** ^1^The Calvin, Phoebe and Joan Snyder Institute for Chronic Diseases, University of Calgary, Calgary, AB, Canada; ^2^Department of Microbiology, Immunology and Infectious Diseases, University of Calgary, Calgary, AB, Canada; ^3^Department of Physiology and Pharmacology, University of Calgary, Calgary, AB, Canada; ^4^Department of Internal Medicine, University of Calgary, Calgary, AB, Canada

**Keywords:** fungal cytotoxicity, tumor cytotoxicity, cytotoxic granules, fungal cytotoxic receptors, fungal cytotoxicity signaling pathways

## Abstract

Natural killer (NK) cells kill or inhibit the growth of a number of fungi including *Cryptococcus, Candida, Aspergillus, Rhizopus*, and *Paracoccidioides*. Although many fungi are not dangerous, invasive fungal pathogens, such as *Cryptococcus neoformans*, cause life-threatening disease in individuals with impaired cell-mediated immunity. While there are similarities to cell-mediated killing of tumor cells, there are also important differences. Similar to tumor killing, NK cells directly kill fungi in a receptor-mediated and cytotoxic granule-dependent manner. Unlike tumor cell killing where multiple NK cell-activating receptors cooperate and signal events that mediate cytotoxicity, only the NKp30 receptor has been described to mediate signaling events that trigger the NK cell to mobilize its cytolytic payload to the site of interaction with *C. neoformans* and *Candida albicans*, subsequently leading to granule exocytosis and fungal killing. More recently, the NKp46 receptor was reported to bind *Candida glabrata* adhesins Epa1, 6, and 7 and directly mediate fungal clearance. A number of unanswered questions remain. For example, is only one NK cell-activating receptor sufficient for signaling leading to fungal killing? Are the signaling pathways activated by fungi similar to those activated by tumor cells during NK cell killing? How do the cytolytic granules traffic to the site of interaction with fungi, and how does this process compare with tumor killing? Recent insights into receptor use, intracellular signaling and cytolytic granule trafficking during NK cell-mediated fungal killing will be compared to tumor killing, and the implications for therapeutic approaches will be discussed.

## Introduction

In the mid-1970s, two independent groups discovered natural killer (NK) cells when they described their ability to lyse tumor cells without prior exposure (1, 2). It is now well established that NK cells are also effective cytotoxic lymphocytes against fungi (3), in addition to their ability to lyse tumors and virus-infected tumor cells (4). NK cell-mediated cytotoxicity is a complex process that involves receptor-mediated binding and signaling, synapse formation, granule polarization, and granule release (5). For tumors, NK cell cytotoxicity is regulated by activating and inhibitory receptors that are expressed on its cell surface. Ligation of these receptors with their cognate ligands triggers downstream signaling events, and a balance in both activating and inhibiting signals tightly controls NK cell function. While there are some similarities with respect to NK cell-mediated killing of tumor cells or fungi, some important differences exist. For example, in tumor cell killing, NK cells release perforin and granzyme B as effector molecules, with the perforin-forming pores in the tumor cell membrane, thereby allowing entrance of granzyme B to activate caspases and induce target cell death (6). In the case of fungi killing, there is no reported use of granzyme B. Instead, NK cells release perforin to directly kill *Cryptococcus neoformans* and *Candida albicans* (7, 8), or granulysin to directly kill *Paracoccidioides brasiliensis* (9), or IFN-gamma to directly damage *Aspergillus fumigatus* (10). NK cells also indirectly mediated fungal elimination by secreting IFN-gamma to either mediate fungicidal activity of murine peritoneal exudate cells against *C. neoformans* (11) or mediate phagocytosis of *C. albicans* by splenic macrophages (12) or secrete GM-CSF to promote neutrophil phagocytosis of *C. albicans* (13). It has also been shown that the receptor and signaling pathway used for cryptococcal killing differs from that used for tumor killing (14, 15). This review will focus on the recent insights into activating receptor-mediated signaling and granule trafficking during NK cell killing of fungi compared to that of tumor cells.

## Receptors Used by NK Cells During Killing

During tumor cytotoxicity, NK cells use a large number of activating receptors to mediate granule-dependent killing. Such receptors include the natural cytotoxic receptors (NCRs) such as NKp30, NKp44, and NKp46, as well as NKG2D, DNAM-1, 2B4, CD2, NKp80, CD48 and Ly9 (CD229), LFA-1, and CD16 (5, 16–28). Interestingly, no activating receptor was found to be sufficient in inducing degranulation, except when used in combination with other receptors (5, 17, 19). It is possible that synergy among NK receptors could be required to mediate fungal cytotoxicity as it is in tumor cytotoxicity.

In the context of fungal cytotoxicity, only two NK cell-activating receptors, NKp30 and NKp46, have been identified. The NKp30 receptor was identified as a fungal cytotoxic receptor when antibodies that were generated against an NK cell line, YT, inhibited fungal killing by NK cells. The NKp30 receptor directly recognized and mediated NK cell killing of *C. neoformans* and *C. albicans* (7) and antibody blocking or siRNA knockdown of NKp30 expression reduced fungal binding and killing (7). More recently, the NKp46 receptor was discovered to directly recognize and mediate killing of *Candida glabrata* (29). NKp46 was identified as a fungal cytotoxic receptor when soluble NKp46–IgG1 fusion construct specifically bounded multiple fungal adhesins, Epa1, 6, and 7, expressed on *C. glabrata* and mediated killing of *C. glabrata* (29). In addition, mice deficient in NCR1 (mouse ortholog of NKp46) could not mediate clearance of systemic *C. glabrata* infection (29). Since several fungi including *Cryptococcus, Candida, Aspergillus*, and *Coccidioides* express adhesins (30), it is interesting to speculate that other fungal adhesins could be recognized by NK cell receptors for fungal cytotoxicity.

## Signaling Pathway Activated in NK Cells During Killing

**Figure 1:**
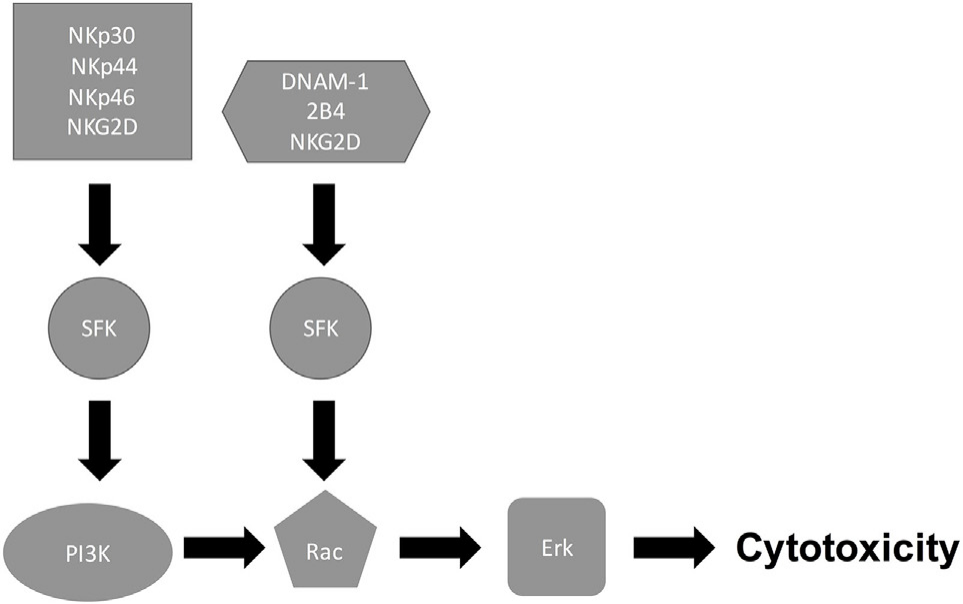
**Natural killer (NK) cell/tumor cytotoxicity pathway**. Following receptor–ligand interaction, NK cell receptors initiate multiple signaling cascades. While the natural cytotoxic receptors (NKp30, NKp44, and NKp46) signal through Src family kinase (SFK) to activate PI3K/Rac/Erk pathway, DNAM-1 and 2B4 signal SFK to activate Rac/Erk pathway, thereby leading to cytotoxicity.

**Figure 2:**
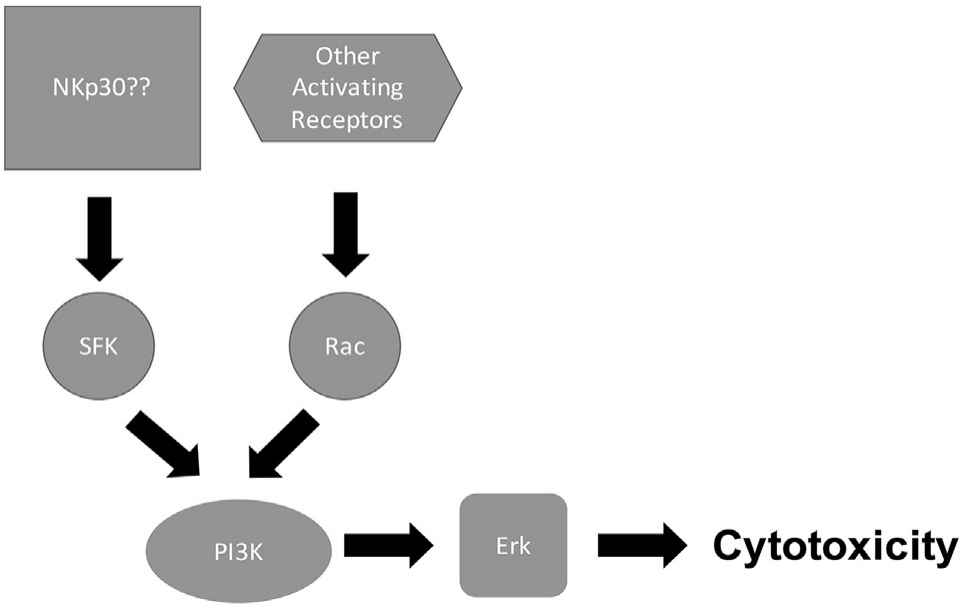
**Natural killer (NK) cell/*Cryptococcus* cytotoxicity pathway**. Following recognition of cryptococcal capsule and cell wall component by NK cell-activating receptors such as NKp30, receptor binding signals both Src family kinase (SFK) and Rac to activate PI3K/Erk, leading to cytotoxicity.

Signaling through NK cell-activating receptors triggers cytotoxicity (6). While the molecular pathways that are associated with NK cell killing of tumor target cells have been elucidated [Figure 1; (6, 31)], the pathways associated with NK cell antifungal activity are still being elucidated (Figure 2). In tumor killing, the inhibition of phosphatidylinositol 3-kinase (PI3K) in NK cells blocked p21-activated kinase 1 (PAK1), MAPK kinase (MEK), and extracellular signal regulated kinase (Erk) activation and interfered with cytotoxic granule movement toward the target cells, thereby suppressing NK cytotoxicity (32). Hence, NK cell antitumor signaling follows the sequential activation of PI3K → Rac1 → PAK1 → MEK → Erk (6, 32). A Vav1 → PLCγ2 → Erk sequence has also been reported to mediate cytotoxicity (31, 33) and Vav1, which is a guanine exchange factor, activated Rac1 by catalyzing GDP/GTP exchange on Rac1 (34). Similar to tumor killing, fungal killing, demonstrated using *Cryptococcus*, depended on PI3K → Erk signaling (35). However, unlike tumor killing, PLCγ was not required for cryptococcal killing (15). Further, in the context of tumor killing, and depending on the activating receptor involved, Src family kinases (SFKs) either directly activated PI3K, leading to Rac and Erk activation, or recruited Vav1 to activate Rac (Figure 1). While the NCRs signal through the PI3K/Rac/Erk axis (6), DNAM-1 and 2B4 signal through the Vav1/Rac/Erk axis (36), and NKG2D transmits its signal through both axes (31, 33, 36–40). Similar to tumor killing, two SFKs, Fyn and Lyn, redundantly mediated NK cell anticryptococcal activity by activating PI3K and Erk1/2 (41). Also, SFK was required to form NK cell-cryptococcal conjugates (15), and NKp30 was required for NK cell–fungal conjugate formation and PI3K–Erk signaling (7), making it likely that NKp30 activated SFK. Unlike in tumor killing where Rac is activated by SFK and is downstream of PI3K, both Rac and SFK were found not to activate each other, but both were essential for PI3K activation in NK cell-mediated killing of *Cryptococcus* (15). In fact, Rac was found to be upstream of PI3K and was required for the activation of the PI3K → Erk signaling pathway during NK cell antifungal activity (15), thereby suggesting two separate pathways for PI3K activation (Figure 2). This interesting finding that Rac is upstream of PI3K needs to be confirmed by others. Ultimately, in tumor and cryptococcal killing, all pathways converged at Erk to mediate granule polarization and exocytosis, resulting in target cell death. The signaling pathway activated by the other known activating receptor for fungi cytotoxicity, NKp46, remains to be identified, and the signaling pathway involved in rearming of fungal cytotoxicity also remains to be investigated (42).

Tumor cells express several activating ligands on their surface to ligate NK cell-activating receptors, thereby resulting in multiple activating signaling pathways that contribute to cytotoxicity. For example, NKG2D binds the ULBP ligands, DNAM-1 binds CD112 and CD155, 2B4 binds CD48, and NKp30 binds B7-H6 (43). To date, the only known activating fungal ligands are three adhesins, Epa1, 6, and 7, of *C. glabrata*, which bind to NKp46 (29). Thus, whether there is coordination of signaling from multiple activating receptors expressed on NK cells to mediate fungi cytotoxicity remains an unanswered question.

## Cytotoxic Granule Trafficking

In response to tumor cells, coordinated signaling through different NK cell receptors and signaling molecules leads to polarization of cytotoxic granules toward the NK immune synapse (NKIS) formed with the target cell (44). The polarization of cytotoxic granules to the NKIS has been described in a sequential order. Upon conjugate formation with the target cell, the engagement of LFA-1 led to Vav1 activation (45). This in turn led to the polymerization and recruitment of filamentous actin (F-actin) to the NKIS (46). Dynein, a minus-end directed motor, rapidly moved granules along microtubules to converge on the microtubule-organizing center (MTOC) (47), followed by the polarization of the MTOC, together with the converged granules, to the NKIS (48). This process was mediated by kinesin-1, a plus-end motor that moves granules in the opposite direction, away from the MTOC (48). Although it is not clear how a kinesin would mediate MTOC movement to the NK cell synapse, in T cell cytotoxicity, the distal microtubule was tethered at the immune synapse and the MTOC was reeled in to the synapse by a dynein motor (49). Also, in T cell-mediated killing, microtubules linked the MTOC to the target contact site and the MTOC was progressively pulled to the contact site by a microtubule sliding mechanism (50). The MTOC movement resulted from the vector sum of tension on multiple microtubules (50). Following polarization, the lytic granules associated with myosin IIA, which enabled their interaction with F-actin and final transit through the actin-rich synapse to join the NK cell membrane (51). The contents of the cytolytic granules were then secreted directly toward the target cell through a pervasive F-actin network at the NKIS (52). While SFK signal mediated the rapid convergence of cytolytic granules to the MTOC without the involvement of PI3K, MEK, or PLCγ (53), PI3K–Erk signal was required for the polarization of the MTOC and converged cytolytic granules to the NKIS (32, 44).

We are only beginning to understand the mechanisms by which cytotoxic granules traffic during fungal killing. Cytotoxic granule trafficking during NK cell cytotoxicity of *Cryptococcus* was different from that of tumors, especially because NK cells did not require LFA-1 in the process, even though the β2 chain of LFA-1 bound to cryptococcal capsular components GXM and GalXM (14). Instead, NK cells used NKp30 to bind *Cryptococcus* and *C. albicans*, mediate microbial synapse formation, and signal PI3K–Erk to release perforin granules (7). Also the SFKs, Fyn and Lyn, redundantly mediated NK cell anticryptococcal activity by activating PI3K and Erk, which in turn polarized perforin-containing granules to the synapse (41). Furthermore, MTOC polarization toward the binding site with *Cryptococcus* was required for cryptococcal killing (54). It remains unknown whether a dynein is required for convergence of granules to the MTOC or whether a kinesin is required for polarization in response to fungi.

## Implication for Therapeutic Approaches

Understanding the receptors used and signaling pathways activated during NK cell function may lead to therapeutic opportunities. For example, compared to healthy adults, NK cells from HIV-infected patients had diminished expression of NKp30 (7), defective binding, reduced perforin content, defective perforin-containing granule polarization (55), reduced perforin release in response to *Cryptococcus*, and reduced cryptococcal killing (7, 55). Interestingly, treatment of NK cells from HIV-infected patients with IL-12 reversed these multiple defects (7, 55). Since a percentage of HIV-infected patients are subclinically infected with *C. neoformans* (56), treatment with IL-12, or similar agent, could reduce or eliminate this complication. Another example is the development of pulmonary cryptococcosis and cryptococcal meningitis in patients with Crohn’s disease or autoimmune hepatitis that were treated with the purine analog, azathioprine (57, 58). Azathioprine prevented Rac1 activation by blocking GTP binding to Rac1 (59, 60), and Rac1 activation in NK cells is required for NK cell cytotoxicity of tumors (32) and *Cryptococcus* (15). Thus, the increased susceptibility to *Cryptococcus* in patients with Crohn’s disease and autoimmune hepatitis that are treated with azathioprine may in part be due to the defective NK cell function resulting from azathioprine-induced blockade of the Rac1 → PI3K → Erk cytotoxicity pathway.

## Concluding Remarks

Fungi, like tumors, are susceptible to NK cell killing. While NK cells use multiple receptors to recognize and kill tumor cells (16), they use NKp30 to recognize and kill *C. neoformans* and *C. albicans* (7), and use NKp46 to kill *C. glabrata* (29). The paradigm in tumor cytotoxicity is that NK cells use multiple activating receptors to recognize tumor targets and the multiple NK cell-activating receptors cooperate to mediate tumor cytotoxicity. Interestingly, several fungi express multiple pattern-associated molecular patterns including adhesins (30), and NK cells used NKp46 to recognize the fungal adhesins Epa1, 6, and 7, which were expressed on *C. glabrata* (30). Further studies are needed to delineate other NK-activating receptors that could mediate fungal killing and to investigate whether cooperative recognition by multiple NK cell-activating receptors is required to mediate fungal cytotoxicity.

## Author Contributions

All the authors listed have made substantial, direct, and intellectual contribution to the work and approved it for publication.

## Conflict of Interest Statement

The authors declare that the research was conducted in the absence of any commercial or financial relationships that could be construed as a potential conflict of interest.
